# A Diffusion-Tensor-Based White Matter Atlas for Rhesus Macaques

**DOI:** 10.1371/journal.pone.0107398

**Published:** 2014-09-09

**Authors:** Elizabeth Zakszewski, Nagesh Adluru, Do P. M. Tromp, Ned Kalin, Andrew L. Alexander

**Affiliations:** 1 Waisman Laboratory for Brain Imaging and Behavior, University of Wisconsin - Madison, Madison, Wisconsin, United States of America; 2 Health Emotions Research Institute, University of Wisconsin - Madison, Health Emotions Research Institute Madison, Wisconsin, United States of America; 3 Department of Psychiatry, University of Wisconsin - Madison, Wisconsin Psychiatric Institute & Clinics, Madison, Wisconsin, United States of America; 4 Department of Medical Physics, University of Wisconsin - Madison, Wisconsin Institutes for Medical Research, Madison, Wisconsin, United States of America; University of Minnesota, United States of America

## Abstract

Atlases of key white matter (WM) structures in humans are widely available, and are very useful for region of interest (ROI)-based analyses of WM properties. There are histology-based atlases of cortical areas in the rhesus macaque, but none currently of specific WM structures. Since ROI-based analysis of WM pathways is also useful in studies using rhesus diffusion tensor imaging (DTI) data, we have here created an atlas based on a publicly available DTI-based template of young rhesus macaques. The atlas was constructed to mimic the structure of an existing human atlas that is widely used, making results translatable between species. Parcellations were carefully hand-drawn on a principle-direction color-coded fractional anisotropy image of the population template. The resulting atlas can be used as a reference to which registration of individual rhesus data can be performed for the purpose of white-matter parcellation. Alternatively, specific ROIs from the atlas may be warped into individual space to be used in ROI-based group analyses. This atlas will be made publicly available so that it may be used as a resource for DTI studies of rhesus macaques.

## Introduction

Brain atlases are essential for investigations of neuroanatomy that are involved in normal and abnormal brain functioning. White matter (WM) atlases based upon diffusion tensor imaging (DTI) may be used to assess differences in structural integrity and connectivity. Atlases of WM structures in humans are widely available and commonly used in studies investigating anxiety disorders [1], depression [2], psychopathy [3], autism spectrum disorder [4], Alzheimer’s disease [5], traumatic brain injury [6] and schizophrenia [7]. For WM analyses of the human brain, Mori and colleagues developed a stereotaxic atlas template of WM structures [8], which herein is referred to as the ICBM-DTI-81 atlas. While modern anatomical atlases with cross-sectional histology and/or MRI scans of the rhesus macaque brain have been created [9]–[11], they focus primarily on gray matter (GM) regions. Much of the pioneering WM mapping work used anatomical dissections [12] and invasive tracers [13] in rhesus monkeys, which have helped to guide WM tractography reconstructions and WM atlases in humans. However, there are currently no available DTI-based WM atlases of the rhesus macaque analogous to the ICBM-DTI-81 in humans.

Diffusion tensor imaging (DTI) is useful for characterizing the microstructure and organization of brain tissues [14], [15]. In WM, the tight fascicle bundles cause the diffusion to be anisotropic, with the direction of greatest diffusivity (the major eigenvector in DTI) corresponding to the local fiber orientation, except in regions of crossing WM tracts. DTI is currently the most widely used neuroimaging method for investigations of WM. However DTI data, like most varieties of MRI data, are challenging to evaluate because the diffusion measures vary considerably across the brain [16]. Therefore, anatomically-specific image analysis strategies, such as region-of interest (ROI)-specific, voxel-based analysis, and tractography-based analyses, must be employed (see [17] for a review). In many of these analytic approaches, brain atlases and templates are needed for defining the anatomical ROIs. Not only are templates derived from DTI scans useful for DTI analyses, but these same ROI templates may also be used for regional measurements using other imaging methods as well.

Because of evolutionarily conserved brain function and structure, the rhesus monkey is a preferred animal model for various neurological and psychiatric illnesses [18], [19], and thus is frequently used in studies of brain structure and function [20], [21]. While *in vivo* surgeries, tracer studies, and *ex vivo* dissections [22]–[27] are often used for mapping structural anatomy and connections, neuroimaging methods are important non-invasive methods for studying the brain *in vivo*. In addition, similar to *in vivo* imaging, comparable techniques can be used in both humans and rhesus monkeys.

While extensive work in the rhesus monkey have been made using histological tracers to map the path of WM fiber bundles, the exact location of tracts is variable among individual subjects and provides only relative references [28]–[30]. For population-based studies that seek to compare quantitative imaging data across multiple subjects, having an atlas that may be registered to each subject is crucial for ensuring the uniformity of regions compared, especially for voxel-based or statistical analyses.

This paper describes the development of a WM atlas based upon a population-averaged DTI template from imaging studies in 271 rhesus macaque monkeys ([31]; http://www.nitrc.org/projects/rmdtitemplate/). We used the design and configuration of the human ICBM-DTI-81 atlas [8] as a guide for manually defining the WM parcellation on the population-averaged monkey DTI template. We also included additional WM parcellation units that covered regions of superficial (near the outer cortex of the brain) and brainstem WM that are not included in the ICBM-DTI-81 atlas. Illustrated brain sections based on anatomical tracer studies of the rhesus [13] were also consulted to identify tracts that were different in shape or proportion from the human atlas. Descriptions of the parcellations are provided and the atlas may be downloaded for applications in research projects focused on the rhesus macaque brain. Finally, the application of the new WM atlas was demonstrated by spatial normalization to a different rhesus brain template created from a different set of animals.

**Figure 1:**
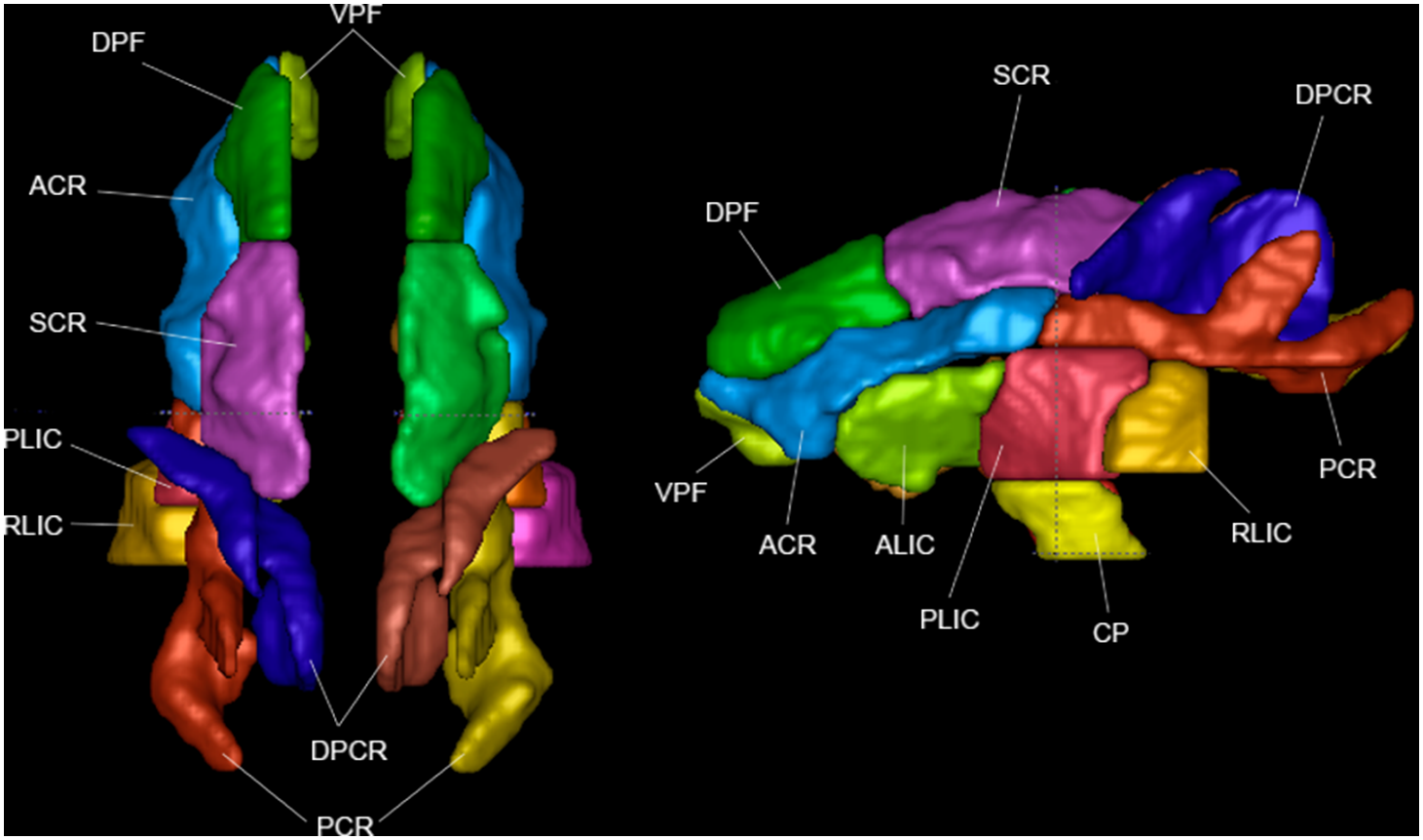
Projection fibers, in top view and left view.

## Methods

The WM atlas was developed using a publicly-available, population-averaged brain template, UW-DTIRMAC271 [31], created from DTI studies from 271 young rhesus macaques in the age range of 0.7370 to 4.2027 years with mean age of 2.4011±0.8795 years. All studies were performed using protocols approved by the University of Wisconsin Institutional Animal Care and Use Committee (IACUC). Details of the image acquisition, DTI processing and template creation are described in Adluru et al. [31]. Briefly, MRI scanning was performed using a GE SIGNA 3T scanner with a 16 cm diameter quadrature birdcage coil and the head was fixed in the sphinx position. DTI scanning was performed using a two-dimensional, echo-planar, diffusion-weighted, spin-echo sequence (TE/TR = 77.2 ms/10 s, field-of-view = 14 cm, 128×128 matrix (interpolated to 256×256 on the scanner), EPI echo spacing = 800 µs) with diffusion weighting at b = 1000 s/mm^2^ in 12 diffusion orientations. A co-planar field map was also obtained using a gradient echo with images at two echo times: TE1 = 7 ms, TE2 = 10 ms. The DWI volumes were eddy-current corrected using FSL [32]. Echo-planar distortions were corrected using a field mapping procedure [33], which was based upon the *fugue* and *prelude* functions in FSL before performing a non-linear tensor estimation [34].

**Figure 2:**
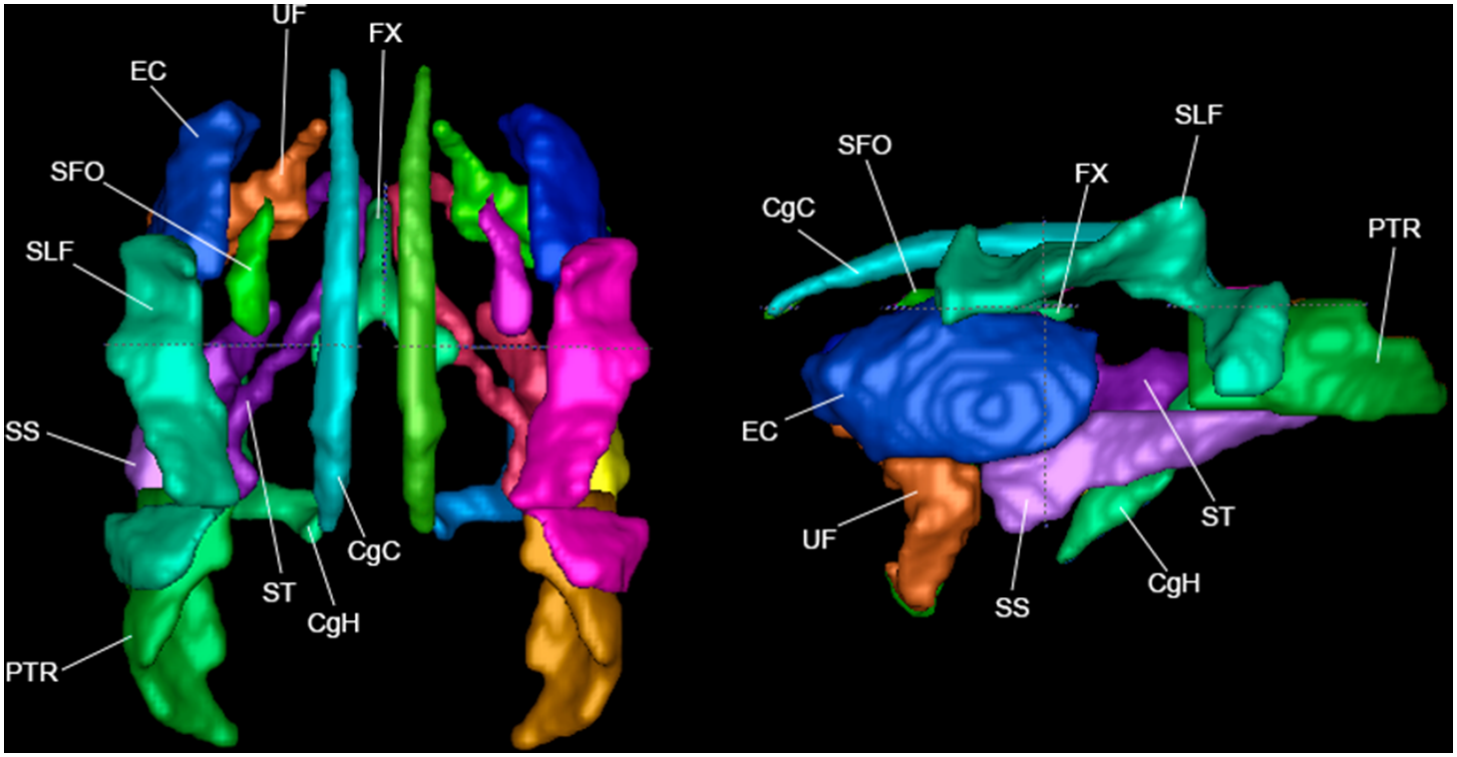
Association fibers in top view and left view.

We registered and resampled the template into the standardized Saleem-Logothetis space [11] with spatial sampling of 0.5 mm×0.5 mm×0.5 mm. Since the anatomy of monkey and human brain white matter is qualitatively similar [35], we used a very carefully delineated and widely used white matter atlas, ICBM-DTI-81 [8] from Johns Hopkins University as a guide. This atlas is available through the FSL brain imaging software package [32]. The UWRMAC-DTI271 template offers an advantage in the task of tracing ROIs, as the template was developed using a tensor-based registration. Since this method preserves full tensor information, as opposed to a scalar based registration, it enabled us to use the directional information via RBG color-coding of directional information to the fractional anisotropy (FA) map. The directional color mapping is often useful for defining boundaries between distinct WM regions. Anatomical features and boundaries including deep GM regions and sulcal definitions were also used to aid in the definition of WM sub-regions. In addition to the 48 regions presented in the ICBM-DTI-81 human atlas, we identified additional ROIs in the brainstem after consulting the Paxinos atlas [10]. Since the ICBM-DTI-81 atlas mainly focused on deep white matter regions, the regions created on our template were expanded to cover the majority of WM in the brain, while consulting a more recent edition of the human atlas [36], [37] as a guide. All regions were first drawn in one plane (often coronal) and repeatedly polished in the axial and sagittal planes to create a smooth and consistent 3D structure.

**Figure 3:**
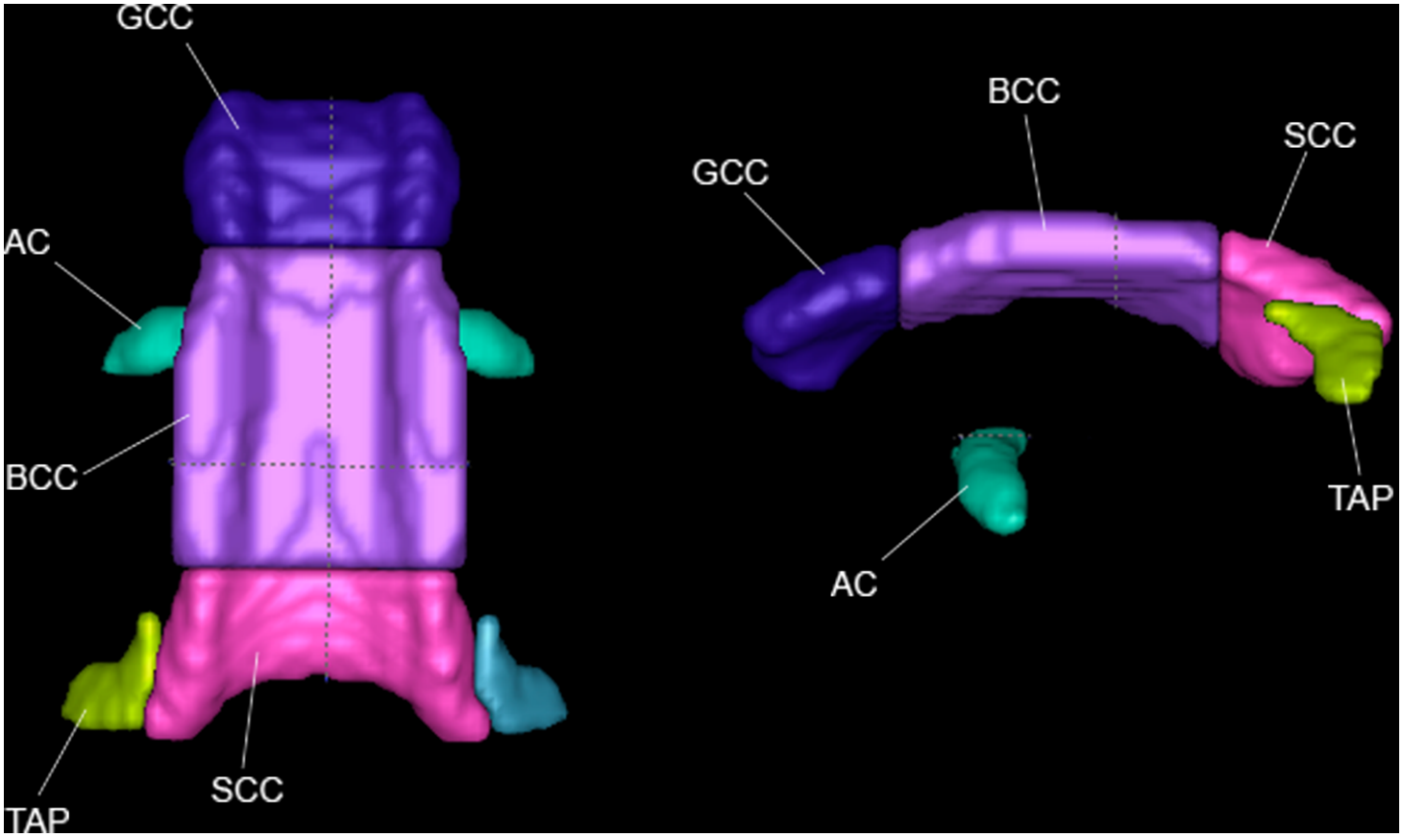
Commissural fibers in top view and left view.

Specific white matter regions, such as the uncinate fasciculus and stria terminalis, were delineated using multi-ROI fiber tracking [38]. The tract reconstructions were used to create a binary mask via rasterization. This mask was used to assist in drawing boundaries of the region for the atlas. To increase the end-user functionality of the atlas, we also made it compatible with a 3D digitized version of the Paxinos [10] atlas, which is integrated with a version of an established T1-weighted (T1w) rhesus template [9]. The WM parcellated atlas was co-registered using an affine transformation to this T1w template using DTI-TK, an advanced DTI spatial normalization and atlas construction tool [39] that also facilitates scalar image registration. This enabled overlay of the WM atlas with the 3D Paxinos atlas, allowing identification of GM and WM ROIs in the same space. In the following sections we define the manual parcellation criteria for each WM region that was identified on the UWRMAC-DTI271 template.

### Projection Tract Regions (Figure 1)

Projection tracts connect the cortex to deep GM regions, such as the thalamus and basal ganglia, and to the spinal cord and cerebellum [40], [41].

**Figure 4:**
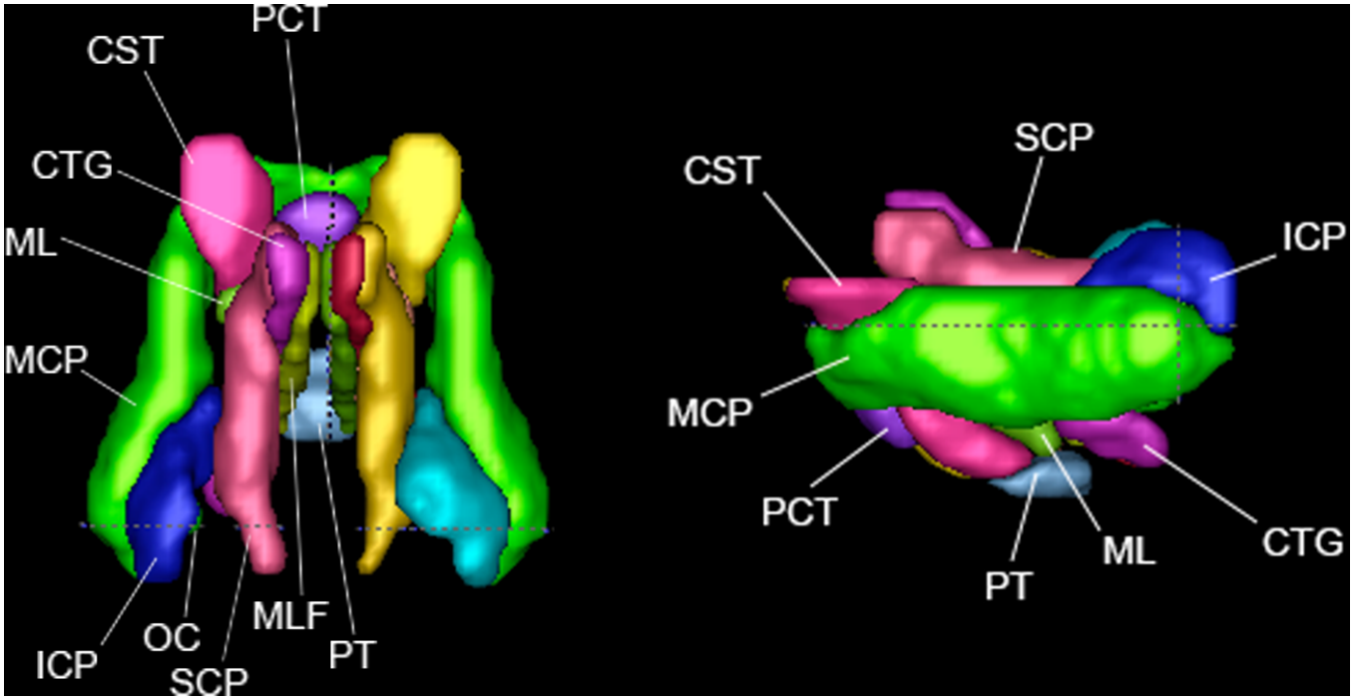
Brainstem fibers in top view and left view.

#### Corona Radiata (CR)

This is a relatively large region that includes pathways between the brainstem and thalamus that project dorsally to the cerebral cortex and its division into subregions is subjective [42]–[45]. The overall regions include the thalamic radiations and parts of the long corticofugal pathways, such as the corticospinal, corticopontine, and corticobulbar tracts [44]. Divisions in this atlas are defined as follows: On the axial level of the corpus callosum are the anterior (ACR) and posterior (PCR) corona radiata, the division happens at a slight change in direction just posterior to the split between the anterior and posterior limbs of the internal capsule. The PCR branches upward in its more posterior portions. Inferior and slightly medial to the ACR lies the ventral prefrontal WM (VPF). Directly superior to the ACR and PCR we define three regions, the dorsal prefrontal WM (DPF), superior corona radiata (SCR), and dorsal posterior corona radiata (DPCR). The division between DPF and SCR occurs roughly where the genu of the corpus callosum becomes the body (BCC). The DPCR begins in a branch of WM lateral to the SCR and gradually moves medially to take its place. It then continues as a sheet of WM located medial to the PCR. The primary orientation of CR tracts is superior/inferior (S/I).

**Figure 5:**
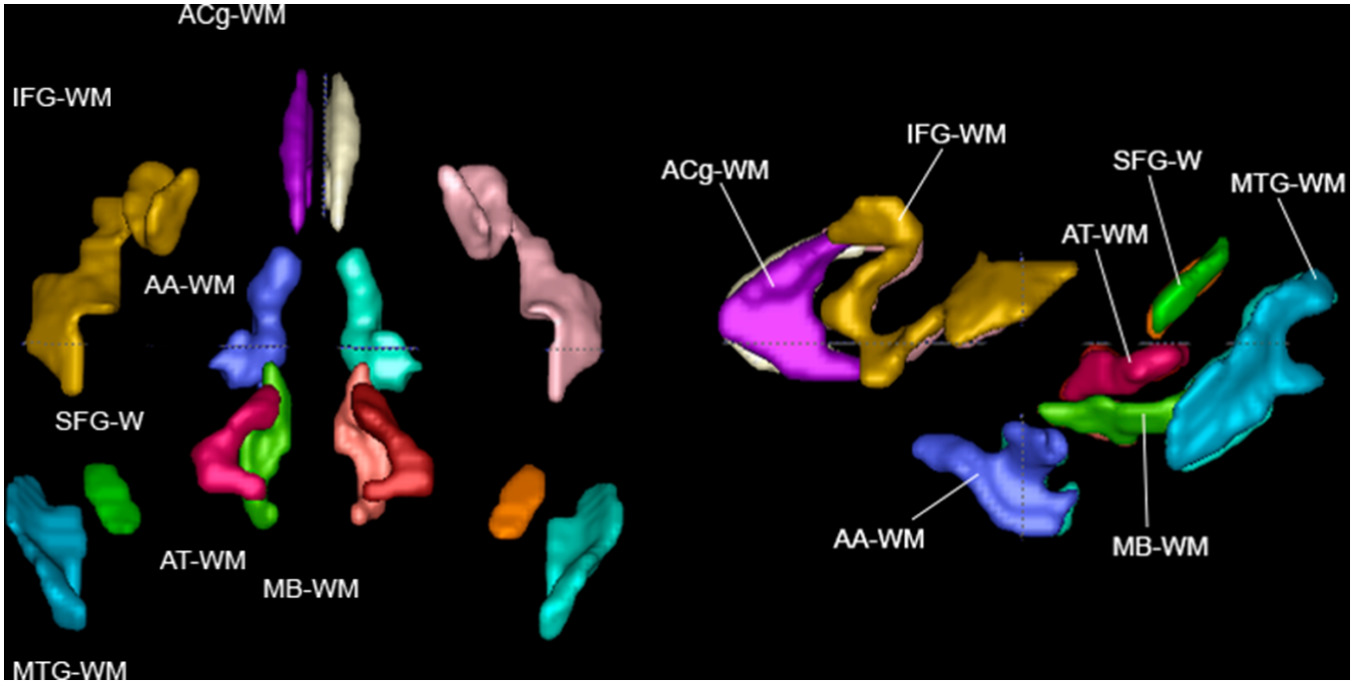
Short range WM regions in top and left view.

#### Anterior Limb of the Internal Capsule (ALIC)

This region consists of thalamocortical fibers and fronto-pontine fibers. These fibers function as part of the medial and basolateral limbic circuits, is involved in information transfer with the prefrontal lobes [46] and have been found to be altered in patients with schizophrenia [47]. The ROI can be identified in axial slices as the part of the internal capsule anterior to the structure’s genu, which contains corticobulbar fibers. The primary orientation of ALIC tracts is anterior/posterior (A/P) and slightly right/left (R/L).

#### Posterior Limb of the Internal Capsule (PLIC)

This ROI contains ascending sensory fibers from the thalamus to the cortex and descending motor fibers of the corticospinal tract (CST). Its fibers are also responsible for conducting sensorimotor signals [46] and injury can cause muscle weakness [48]. It contains the corticospinal, corticopontine, and corticobulbar tracts. It is defined in axial slices as the part of the internal capsule posterior to the structure’s genu. The primary orientation of PLIC tracts is S/I and slightly R/L.

#### Retrolenticular Limb of the Internal Capsule (RLIC)

This ROI contains reciprocal connections between the lateral geniculate nucleus and occipital lobe, including the optic radiation. Its fibers are also responsible for motor function. It is defined in axial slices as beginning anteriorly at the point the PLIC and external capsule join, and posteriorly it is defined as ending (or becoming the posterior thalamic radiation) at the point where the fibers begin to curve in a more lateral direction. The primary orientation of RLIC tracts is A/P.

#### Cerebral Peduncle (CP)

This region contains fibers that connect the cerebrum to the brainstem and extends between the PLIC superiorly and the corticospinal tract inferiorly. Its fibers function largely in motor control and it also caries some fibers from the prefrontal cortex (in the macaque; the proportions are reversed in the human) [49]. It contains corticospinal, corticopontine, and corticobulbar tracts, and makes up the majority of the midbrain. The primary orientation of CP tracts is S/I.

### Association Tract Regions (Figure 2)

Association tracts connect different parts of the cortex within the same hemisphere.

#### Superior Longitudinal Fasciculus (SLF)

This region includes tracts that lie laterally to portions of the corona radiata and external capsule. It include tracts that connect the frontal, parietal, occipital, and temporal lobes, that are important in language processing [50], [51]. The primary orientation of tracts in the SLF is A/P, except in the posterior region where it runs S/I. In this atlas, we define a more extensive region to be covered by the SLF than in the human ICBM-DTI-81 atlas, which is supported by other tractography studies [52], but the region is still small enough that we did not attempt to divide it into SLF I, SLF II, and SLF III which have been described in monkeys [53].

#### Superior Fronto-Occipital Fasciculus (SFO)

This ROI is located just medial to the external capsule and anterior corona radiata, and superior to the anterior limb of the internal capsule (farther posterior it runs medial to the ALIC). The SFO tract is used in language processing, in particular the initiation and preparation of speech movements [54], [55]. It is a historically hard-to-define tract due to its proximity to the corona radiata and its subcallosal location. The primary orientation of the SFO is A/P, though the superior portion is more S/I.

#### Uncinate Fasciculus (UNC)

This region corresponds to a fishhook shaped tract that connects the frontal lobe and the anterior and medial temporal lobe, stretching inferior and posterior from the frontal lobe and then curving at the temporal lobe to run anterior again. The UNC region was defined using ROI-based streamline tractography using inclusion ROIs in the temporal lobe and prefrontal lobe defined on coronal sections. Note that the UNC definition here is much more extensive than for the human ICBM-DTI-81 atlas. The primary orientations of the UNC are A/P and S/I.

#### Sagittal Striatum (SS)

This region is defined here to include a large part of both the inferior longitudinal fasciculus (ILF) and inferior fronto-occipital fasciculus (IFO). It projects to the frontal and limbic cortices [56], [57]. It begins axially in the slice just posterior to the bend of the UNC, and continues posteriorly to occupy the space just inferior to the external capsule, then the RLIC, and finally the posterior thalamic radiation. The primary orientation of the SS is A/P.

#### External Capsule (EC)

This region is located lateral to the internal capsule, separated by the globus pallidus and putamen. It includes cortical projections from the corpus callosum to the striatum [58], with an auditory pathway, which causes paraphasia when lesioned [59]. It is easily defined with its gentle lateral curve as it runs in the superior-inferior direction across many axial slices. The region may also contain some fibers of the extreme capsule that cannot be distinguished at this resolution. The primary orientation of the EC is A/P, curving slightly in other directions in places.

#### Superior Cingulum (CgC)

The cingulum bundle region includes an association fiber tract that is part of the limbic system and connects medial pericallosal brain regions (i.e., cingulate gyrus) to the hippocampus and amygdala. The CgC region runs predominately anterior/posterior and curves above the medial parts of the corpus callosum between the anterior cingulate gyrus to the precuneus. The primary orientation of CgC is A/P.

#### Perihippocampal Cingulum (CgH)

This region is a continuation of the CgC from the precuneus to the hippocampus. The region curves ventrally and anteriorly along a medial path towards the hippocampus. As a connection of the cingulum and hippocampus, it contains fibers that play an important role in attention and memory retrieval. The primary orientation of CgH tracts is a combination of A/P and S/I.

#### Posterior Thalamic Radiation (PTR)

This region includes fiber pathways that connect the caudal parts of the thalamus with the occipital lobe and parietal lobe [60], and are important for sensorimotor function such as touch and proprioception. The ROI begins at the posterior edge of the RLIC (a boundary chosen to roughly coincide with the anterior edge of the inferior portion of the tapetum), and continues to extend to the far posterior reaches of white matter in the occipital lobe. The primary orientation of PTR tracts is A/P.

#### Fornix (FX)

This region, located just below the body of the corpus callosum (CC), is a part of the limbic system important to memory function and a main efferent of the hippocampus [61], connecting it to the hypothalamus and other subcortical structures. The fornix is a single fiber bundle across the midline in its anterior section, above the anterior commissure (AC), and branches into two parts moving laterally into the hemispheres in its posterior portion. The primary orientation of FX is S/I at the midline and A/P in the branching section.

#### Stria Terminalis (ST)

This region is localized to a limbic tract, which carries information from the amygdala to the septum and hypothalamus, is often defined as a part of the fornix [8]. The region spans from the posterior amygdala and runs superiorly and anteriorly over the thalamus, in the form of a ring encircling the internal capsule, toward the bed nucleus of the stria terminalis [BNST] [62]. By connecting the amygdala and BNST, this tract is important in conveying information involved in regulating stress, fear, and anxiety [63]. The primary orientation of the ST changes throughout its curved path. Because of its complicated path, the ROI was defined with the help of a tractography-based mask.

### Commissural Tract Regions (Figure 3)

The commissural tracts connect the two hemispheres of the brain.

#### Anterior Commissure (AC)

This region contains a transversely running bundle of white matter fibers that crosses the midline and runs right-left connecting the cerebral hemispheres. The tracts transmit multisensory messages between hemispheres [64]–[66], and connect the anterior and ventral temporal lobes [67]. It begins medially just below the genu of the internal capsule and in front of the anterior columns of the fornix. It then curves slightly more inferiorly as it extends laterally into the hemispheres. The primary orientation of the AC is R/L.

#### Corpus Callosum (CC)

The majority of fibers that cross the midline pass through the corpus callosum, and it is one of the most easily identified WM structures. It mainly provides connections between homologous cortical areas in both hemispheres, and mediates the interhemispheric communication among cortical areas underpinning higher-level cognitive function [68], [69]. Here the coronal boundaries between the genu (GCC), body (BCC), and splenium (SCC) are made to roughly match the human ICBM-DTI-81 atlas. The primary orientation of the CC is R/L.

#### Tapetum (TAP)

This small structure extends the posterior portion of the SCC laterally and inferiorly. It lies just medial to the anterior portion of the posterior thalamic radiation. It is the temporal component of the CC [8], and connects the temporal lobes [70], [71], possibly assisting with sensory integration and working memory. The primary orientation of this portion of TAP is R/L.

### Brainstem White Matter Regions (Figure 4)

Brainstem tracts carry information between peripheral nerves and the spinal column and other parts of the brain, and they pass through the brainstem.

#### Corticospinal Tract (CST)

The CST includes pathways that connect the motor areas of the cortex (along with some parietal areas) to the spinal GM, and serves to modulate motor functions [72]. As in the ICBM-DTI-81 atlas, this region is identified at the level of the pons and extends dorsally from the pyramidal tracts (most inferior part of brain) in more posterior coronal slices until meeting with the cerebral peduncle. It also contains the corticobulbar and corticopontine tracts. The primary orientation of the CST is S/I.

#### Medial Lemniscus (ML)

Located directly posterior to the pontine tracts, this ROI originates from just superior to the pyramidal tracts, where it lies directly posterior to the CST, and passes superiorly directly anterior to the central tegmental tract. It stops before reaching the midbrain level, where it meets the superior cerebellar peduncle. It contains the core of a major sensory pathway that serves to connect the gracile and cuneate nuclei to the ventral posterolateral nucleus of the thalamus [73] and is a somatosensory pathway with a primary function to convey discriminative touch, vibration, and conscious proprioception from the trunk and extremities [74]. The primary orientation of the ML is S/I and slightly A/P in the superior end.

#### Medial Longitudinal Fasciculus (MLF)

This ROI lies in the medial part of the brainstem, and ascends while moving more anterior (inferior to the superior cerebellar peduncle) before lying just inferior to the central tegmental tract. The fiber tract helps to control eye movement and also connects to motor neurons in the lower cervical spinal cord [75]. The primary orientation of MLF tracts is between S/I and A/P.

#### Inferior Cerebellar Peduncle (ICP)

This region includes tracts that carry information from the spinal cord and medulla (beginning just superior to the central tegmental tract) to the cerebellum as it passes superiorly and curves around anterior and superior to the olivocerebellar tract. Its function is integrating proprioceptive sensory input with motor vestibular functions such as balance and posture maintenance [41]. The primary orientation of the ICP is S/I.

#### Middle Cerebellar Peduncle (MCP)

This ROI includes tracts that initiate from the pontine nuclei and carry information between the cortex and the cerebellum. It is concerned with proprioception and sensory afferent input [76], [77]. At the coronal level of the pons it begins just below the CST, and extends posteriorly to the cerebellum. The primary orientation of the MCP is A/P, curving R/L at its anterior end.

#### Superior Cerebellar Peduncle (SCP)

This ROI begins on the coronal slice just before the MCP first divides into bilateral tracts, and continues posteriorly running first above the pyramidal fibers and moving laterally toward the top of the cerebellum. The tract is important for motor control [78]. The ROI contains axons that carry information from the deep cerebellar nuclei to the thalamus. The primary orientation of the SCP is A/P.

#### Central Tegmental (CTG)

This ROI represents a compact bundle of somatosensory fibers [79] that occupies a large triangular area lateral to the MLF; posteriorly it descends to the olivary nucleus. It also includes numerous fibers ascending from the medullary, pontine, and mesencephalic reticular formation to the thalamus and subthalamic region [80]. The primary orientation of the CTG is between S/I and A/P.

#### Olivocerebellar (OC)

This region includes a small group of fibers that originate at the olivary nucleus and have projections to the cerebellum. Its function is to forward somatosensory information between the spinal cord and the cerebellum [81]. This ROI lies just medially to the ICP. The primary orientation is mostly A/P and slightly R/L.

#### Pyramidal Tracts (PT)

These regions lie in the most inferior region of the brainstem that can be resolved in a skull-stripped image. The ROI is defined here as a continuation of the CST bilaterally, and proceeds posteriorly joining across the midline to form one tract. The PT carry axons that originate in the motor cortex and terminate in the spinal cord, and are often defined as a part of the CST and/or corticobulbar tracts (the later not having its own definition here). The primary orientation of PT is A/P.

#### Pontine Crossing Tract (PCT)

A part of the MCP, this region is named because it crosses the midline at the level of the pons. It makes this crossing medial to the CST, and splits bilaterally posteriorly to terminate below the ML [30]. The primary orientation of the PCT is R/L.

### Short Range WM (Figure 5)

The regions based on the ICBM-DTI-81 human atlas were extended to include the majority of WM in the brain. Subcortical regions were added and named based on a GM structure from Paxinos [10] to which that region is adjacent. Peripheral WM regions were added and named based on nearby anatomical landmarks in a similar fashion to an extended human atlas [36]:

#### Inferior Frontal Gyrus WM (IFG-WM)

This region consists of WM in the inferior frontal gyrus, lateral to the DPF and the SLF, following the convolutions of the gyri. The primary orientation of most IFG-WM tracts is R/L (part of the tract is A/P).

#### Superior Temporal Gyrus WM (STG-WM)

The small area of the WM of the superior temporal gyrus is in the next gyrus posterior to the IFG-WM, and lies laterally to the RLIC and, more superiorly, the SLF. The primary orientation of STG-WM tracts is R/L.

#### Middle Temporal Gyrus WM (MTG-WM)

The WM of the middle temporal gyrus is in the next gyrus posterior to the STG-WM, and lies laterally to the SS and, more superiorly, the SLF. The primary orientation of most MTG-WM tracts is between R/L and A/P (the most inferior portion has fibers that curve S/I).

#### Adjacent Thalamus WM (AT-WM)

The WM in the thalamus area is defined starting medial to the ST, PLIC, and superior portion of the CP, resting just superior to the midbrain. The primary orientation of AT-WM tracts is R/L.

#### Midbrain White Matter WM (MB-WM)

This region covering WM in the vicinity of the midbrain is defined inferior to the AT-WM in the space medial to the CP. Its most inferior portion ends just anterior to the SCP. The primary orientation of most MB-WM tracts is A/P.

#### Adjacent Amygdala White Matter (AA-WM)

The WM in the area of the amygdala begins inferior to the anterior-most part of the ST. Moving posteriorly, it remains medial to the anterior end of the ST, and then stretches inferiorly down towards the temporal lobe. Its superior portion then stretches laterally and eventually touches the posterior end of the ST. This area may include fibers of the temporoparietopontine tract and/or the optic tract. The primary orientation of these tracts is A/P with parts oriented R/L.

#### Anterior Cingulum WM (ACg-WM)

This region consists of WM in the area superior to and slightly medial to the CgC. It extends inferior of the CgC just medial to the ventral prefrontal WM. The primary orientation of anterior cingulum WM tracts is R/L.

### Evaluation of automatic segmentation of WM regions by comparison to manual segmentation

To assess the consistency of this atlas to other DTI data, the UWRMAC-DTI271 template was spatially normalized to a DTI brain template from a different rhesus macaque study (unpublished). This study consisted of 22 subjects (mean age of 1.79±0.65 years) that were scanned twice. The template of this study was also created using DTI-TK’s diffeomorphic tensor normalization tools. The template FA maps from both studies were co-registered using ANTS [82] spatial normalization software. The WM regions defined on the UWRMAC-DTI271 template were spatially transformed onto the new template and compared to manual segmentation of three regions, which we designated as the “gold standard”. We evaluated three tracts - the body of the corpus callosum (BCC), the anterior limb of the internal capsule (ALIC), and the uncinate fasciculus (UNC). The overlap of the manually defined regions on the new template and the transformed UWRMAC-DTI271 regions was evaluated using the Dice coefficient (D), a measure of overall agreement between the two volumes, as defined by:
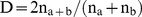
(1)where n_a+b_ is the number of voxels occurring in sets a and b (the intersection), n_a_ is the number of voxels in set a and n_b_ is the number of voxels in set b. A Dice coefficient of 1 would indicate perfect agreement, and 0 indicates no overlap.

## Results

**Figure 6:**
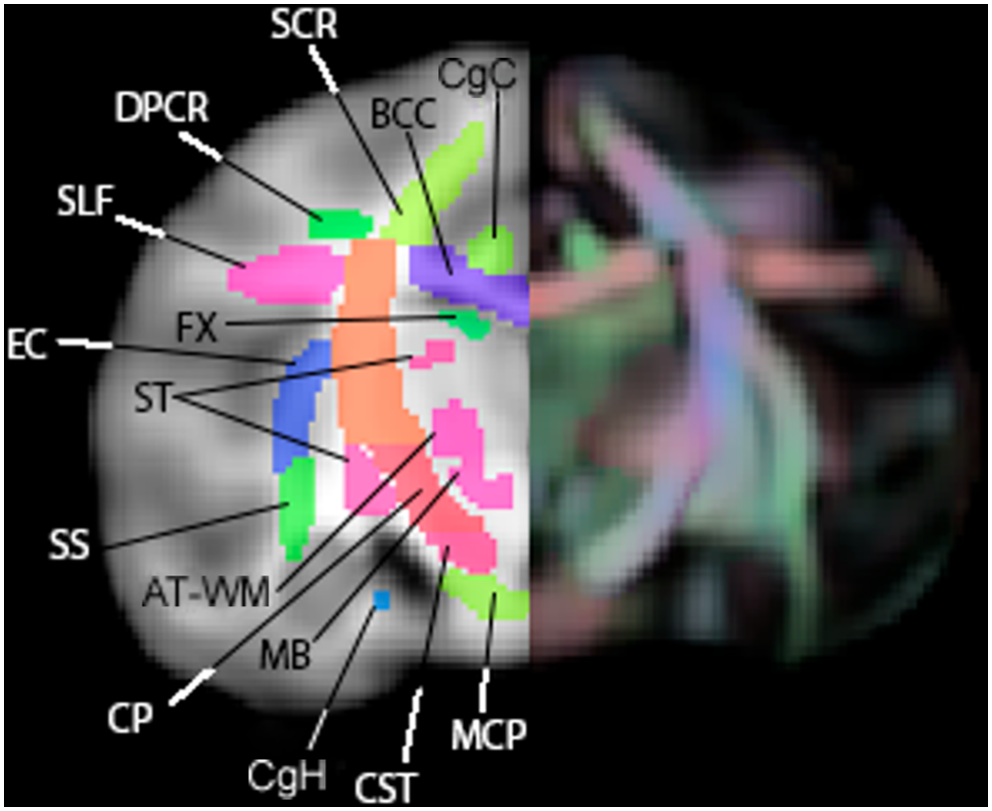
A coronal slice of the WM atlas. Overlaid on a T1-W template (left) and an FA color map (right). AT-WM = adjacent thalamus, BCC = body of corpus callosum, CgC = superior cingulum bundle, CgH = perihippocampal cingulum tract, CP = cerebral peduncle, CST = corticospinal tract, DPCR = dorsal posterior corona radiata, EC = external capsule, FX = fornix, MCP = middle cerebellar peduncle, SCR = superior corona radiata, SLF = superior longitudinal fasciculus, SS = sagittal striatum, ST = stria terminalus.

**Figure 7:**
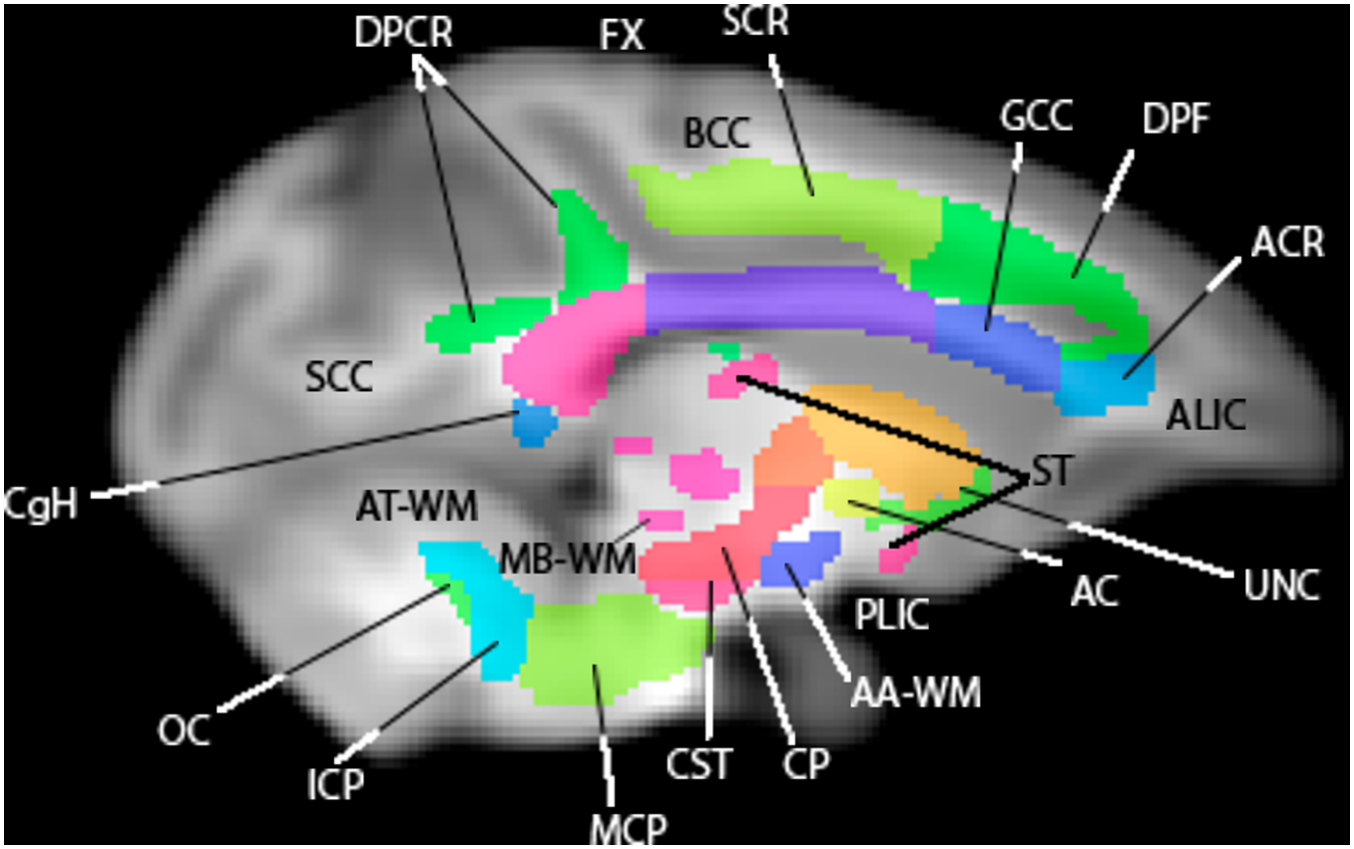
A sagittal slice of the WM atlas. Overlaid on a T1-W template. AC = anterior commissure, AT-WM = adjacent thalamus, AA-WM = adjacent amygdala, ALIC = anterior limb of the internal capsule, BCC = body of corpus callosum, CgH = perihippocampal cingulum tract, CP = cerebral peduncle, CST = corticospinal tract, DPCR = dorsal posterior corona radiata, DPF = dorsal prefrontal, FX = fornix, GCC = genu of corpus callosum, ICP = inferior cerebellar peduncle, MB-WM = midbrain, MCP = middle cerebellar peduncle, OC = olivocerebellar tract, PLIC = posterior limb of the internal capsule, SCC = splenium of corpus callosum, ST = stria terminalis, UNC = uncinate fasciculus.

**Figure 8:**
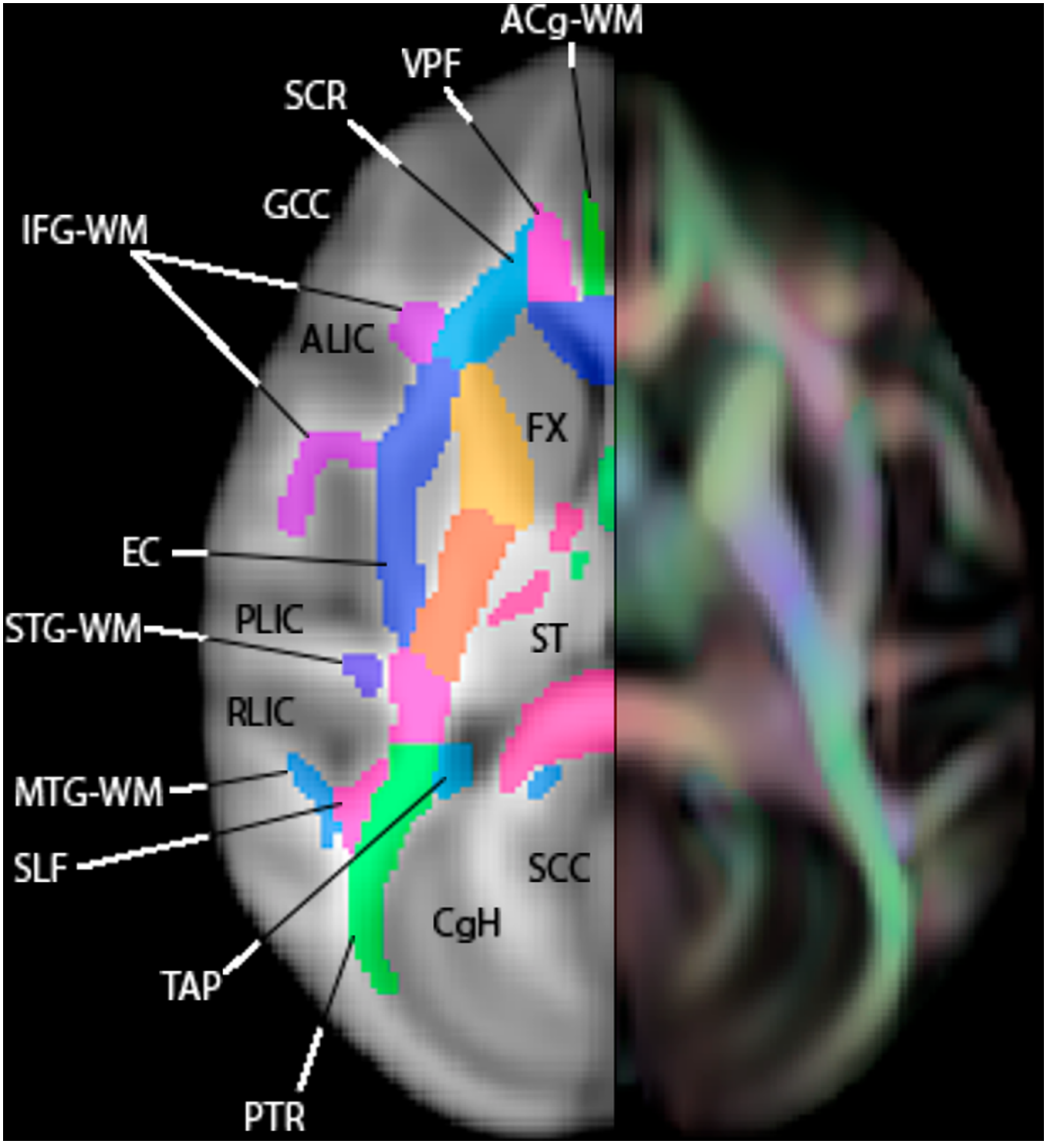
An axial slice of the WM atlas. Overlaid on a T1-W template (left) and an FA color map (right). ACg-WM = anterior cingulum, ALIC = anterior limb of the internal capsule, CgC = superior cingulum bundle, CgH = perihippocampal cingulum tract, EC = external capsule, FX = fornix, GCC = genu of corpus callosum, IFG-WM = inferior frontal gyrus WM, MTG-WM = middle temporal gyrus WM, PLIC = posterior limb of the internal capsule, PTR = posterior thalamic radiation, RLIC = retrolenticular limb of the internal capsule, SCC = splenium of corpus callosum, SCR = superior corona radiata, SLF = superior longitudinal fasciculus, ST = stria terminalus, STG-WM = superior temporal gyrus WM, TAP = tapetum.

**Figure 9:**
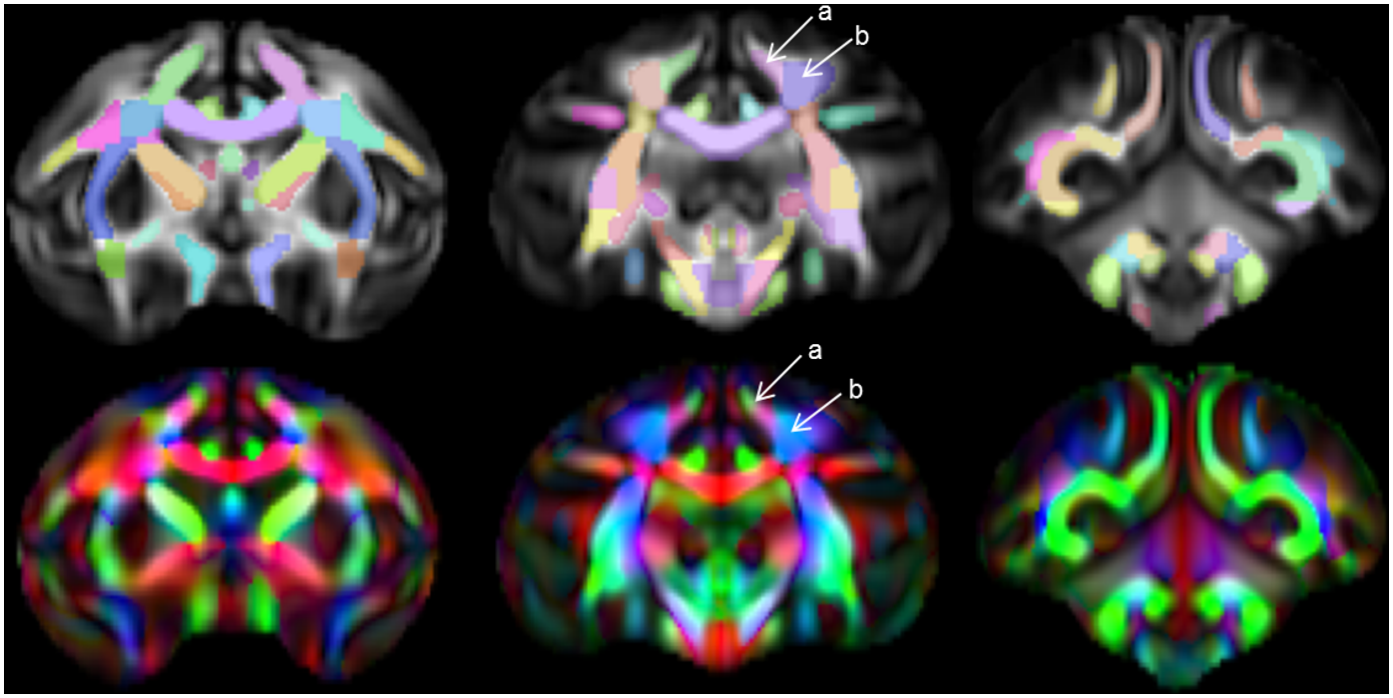
Delineation between tracts. Top: FA maps of select slices of template (anterior on left, posterior on right) with atlas ROIs overlaid. Bottom: Color FA map of matching slices, showing delineation between tracts. Arrows indicate the division between the SCR (a) and the DPCR (b), and the directional (color) changes that guided the decision of where to put this boundary.

The atlas contains 76 labeled regions (including both bilateral regions and a few inter-hemispheric single regions. Figures 6, 7, and 8 show the WM atlas overlaid on a T1w-weighted standard template [9] in coronal, sagittal, and axial views, respectively. In figures 6 and 8, the T1w-weighted image is shown on the left, alongside the FA directional color map from the UWRMAC-217 DTI template [31] on the right. All structures in these displayed slices of the atlas are labeled. The structures cover all of the major high-FA WM regions and even some structures that are too faint to be seen in the color map. To make it more clear how the ROIs relate to the template FA on which they were drawn, several sagittal slices are shown in Figure 9. By looking at the color map, one can tell, for example, how the border between the SCR (a) and DPCR (b) was selected.

**Figure 10:**
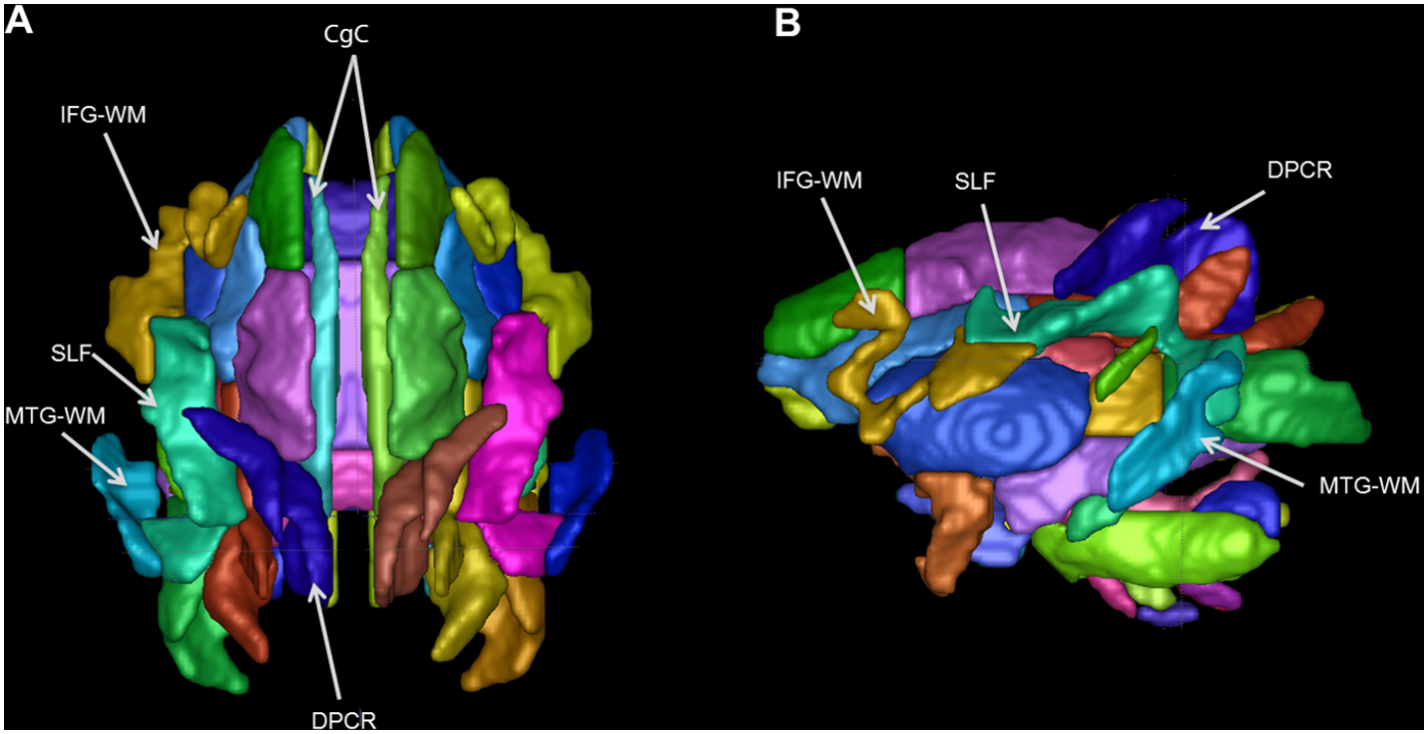
3D renderings of atlas. (A) Superior 3D surface rendered view of atlas. (B) Left 3D surface rendered view of atlas. The superior cingulum bundle (CgC) and many peripheral WM tracts are pointed out. IFG-WM = inferior frontal gyrus WM, SLF = superior longitudinal fasciculus, MTG-WM = middle temporal gyrus WM, DPCR = dorsal posterior corona radiata.

Figure 10 shows a 3D view of the atlas alone, which makes the shapes of the structures more clear. Some shapes are easily recognizable, such as the cingulum bundles (CB). The more convoluted shapes of the SLF and other peripheral WM tracts are more easy to see here than from written description.

**Figure 11:**
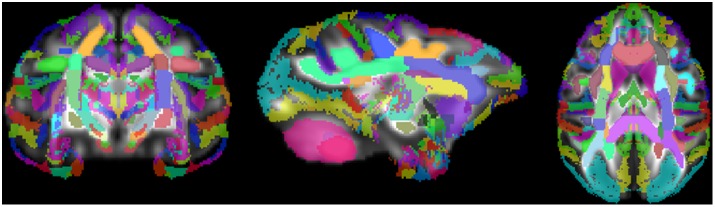
WM atlas and digital Paxinos atlas of GM regions overlaid on UWRMAC-DTI271 template FA map.

Since the template was able to be registered to the McLaren atlas [9], in which space the 3D Paxinos atlas was created, we could overlay both atlases at once. The result is a 3D atlas that covers both the cortical and subcortical GM structures (Paxinos) and all the WM tracts. As can be seen in Figure 11, the two atlases together cover the majority of space in the rhesus brain. While there may be some small overlap between GM and WM structures, we do not see this as being an issue for most applications. For studies in which one is interested in comparing certain WM and certain GM structures across populations, this allows the study’s template to be registered once, to one atlas space, to identify any ROIs that are needed. By avoiding an extra transformation, one can be spared of the data loss that may occur during image registration, and improve the statistical validity of the overall comparisons in the study.

**Figure 12:**
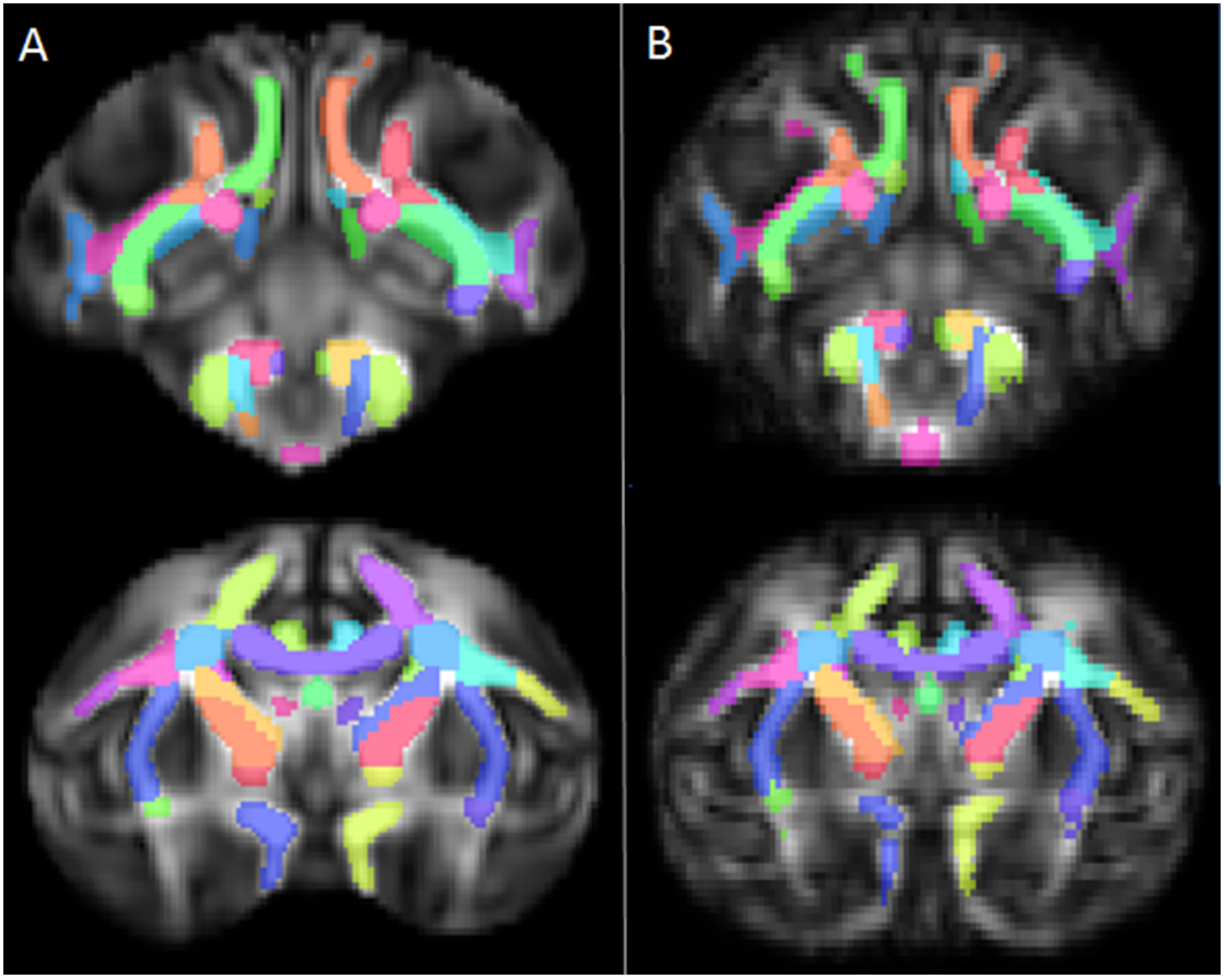
Registering to a different template. Two representative coronal slices of the atlas in its default space (A) and after registering with nearest-neighbor interpolation to a template made from a smaller number of young rhesus macaques (B), which were not a part of the original DTI template used to draw the atlas. White matter coverage is equally good in the registered atlas, qualitatively.

**Figure 13:**
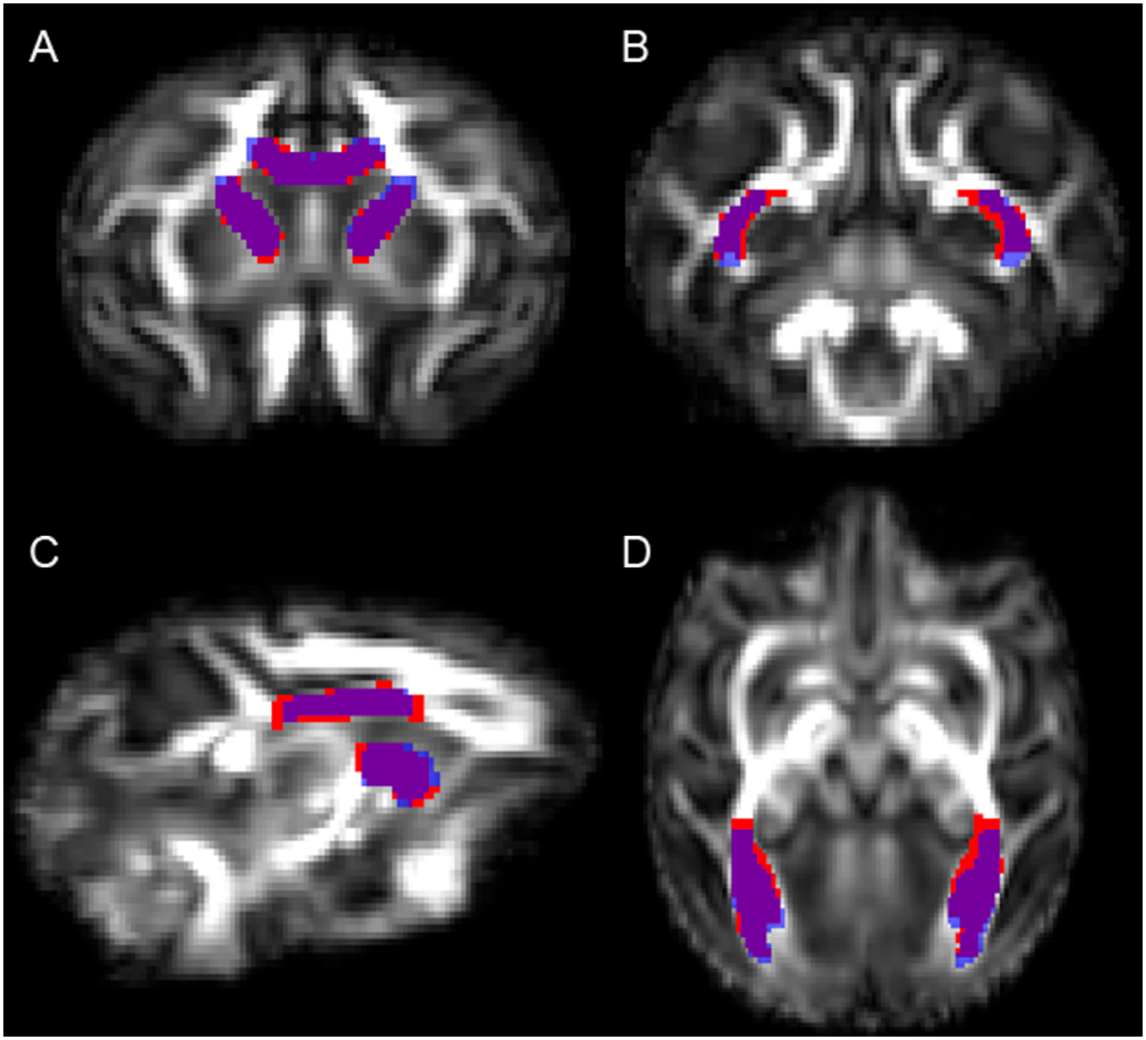
Image showing overlap between manual (blue) and automatic (red) methods of defining tracts. Manual definitions of the corpus callosum and ALIC (anterior limb of the internal capsule (A, C) and the PTR (posterior thalamic radiation) (B, D) were drawn on the different template and compared to automatically defined regions. Overlap agreement appears in purple. Slices were selected that illustrated areas where the two segmentation methods disagreed.

The atlas registration to the new rhesus template is shown in Figure 12. The overlap of the manually selected and atlas-defined regions are visualized in Figure 13 and the Dice coefficient measures of overlap are listed in Table 1. The first three columns contain the values of n_a+b_, n_a_, and n_b_, respectively. The fourth column contains the value of the Dice coefficient (D, Eq. 1) for each of the regions. The “bilateral” regions of the posterior thalamic radiation (PTR) and anterior limb of the internal capsule (ALIC) are simply the left and right regions combined into one image. To test for consistency, the average of the Dice coefficients for the left and right regions is displayed in the fifth data column, next to the Dice coefficient of the actual combined bilateral region.

**Table 1:** Agreement between automated and hand segmentation in the BCC (body of corpus callosum), PTR (posterior thalamic radiation) right, left, and bilateral, and ALIC (anterior limb of the internal capsule) right, left, and bilateral.

	Voxels in overlapregion	Voxels total inautomatedsegmentation	Voxels total in handsegmentation	Dice coefficient	Average Dice coefficient in left and right regions
**BCC**	2427	3169	2573	**0.84535**	
**Left PTR**	1068	1480	1334	**0.759062**	
**Right PTR**	1043	1344	1286	**0.793156**	
**Bilat. PTR**	2111	2824	2620	**0.775532**	0.776109
**Left ALIC**	806	955	959	**0.842215**	
**Right ALIC**	864	1087	983	**0.834783**	
**Bilat. ALIC**	1670	2042	1942	**0.838353**	0.838499

**Figure 14:**
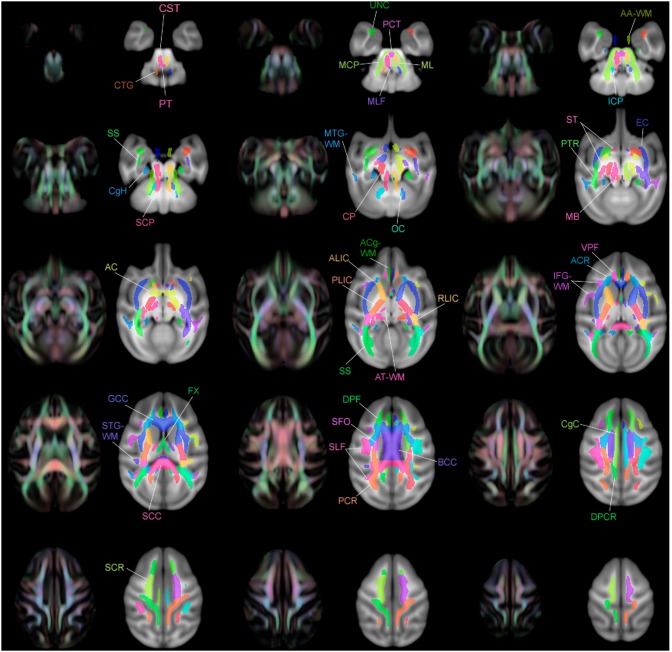
Multiple slices of atlas overlaid on T1-W template beside directional FA color image, axial view.

**Figure 15:**
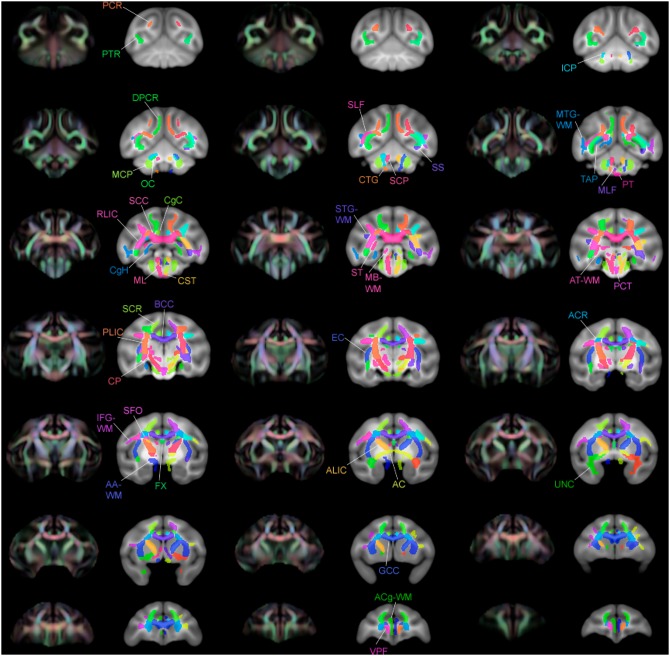
Multiple slices of atlas overlaid on T1-W template beside directional FA color image, coronal view.

**Figure 16:**
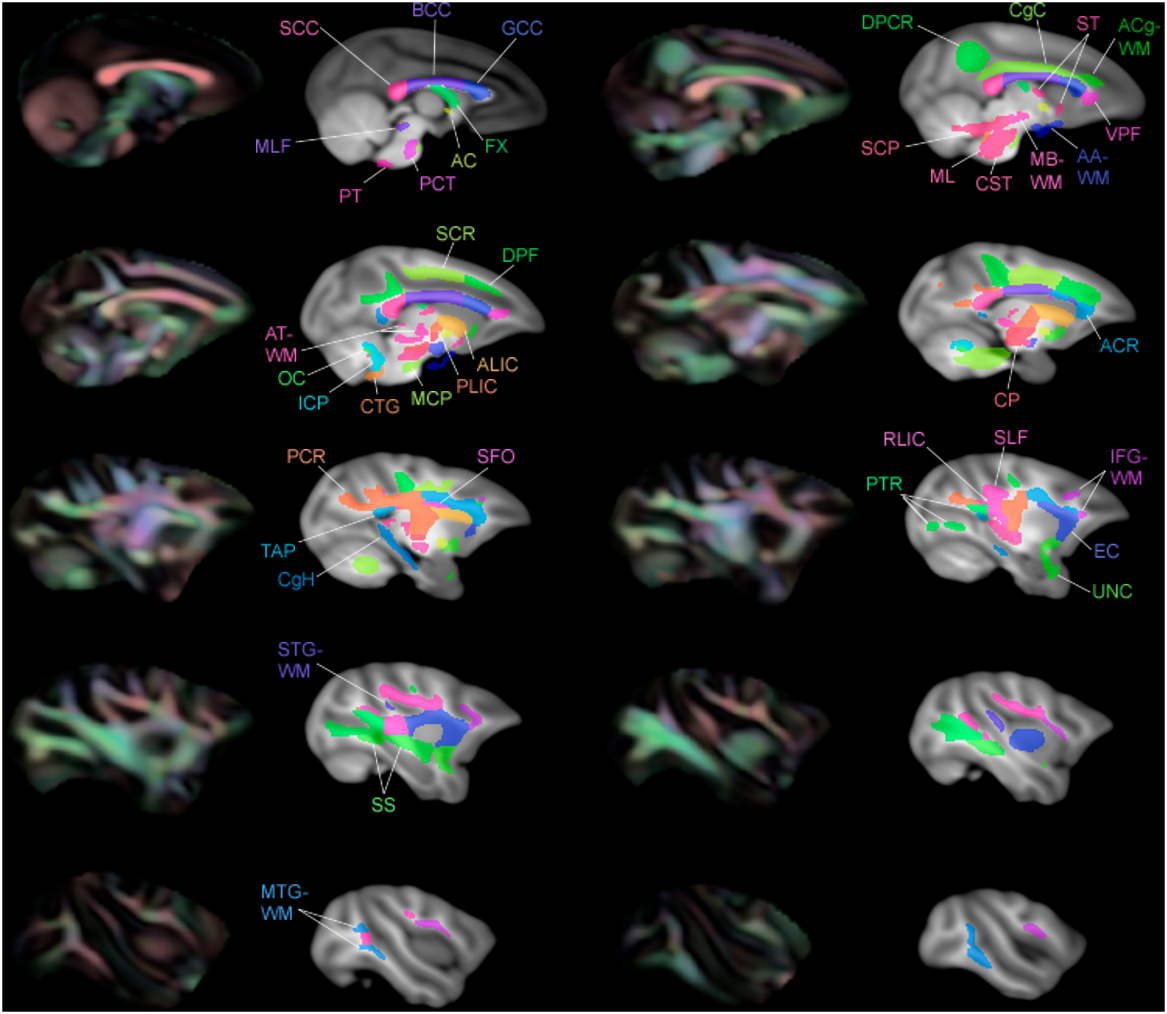
Multiple slices of atlas overlaid on T1-W template beside directional FA color image, sagittal view.

Multiple views of select slices of the atlas covering the whole brain are shown in Figures 14, 15, and 16. Each structure in the atlas is labeled once in each figure in a slice where it is prominent. Abbreviations correspond to those used in the Methods section.

## Discussion

The rhesus macaque is a species that lacks the number of well-organized MRI-based templates that are found in humans. While many worthy attempts at rhesus MRI atlases have been made in recent years [9], [83]–[85], the focus has remained on cortical and deep gray structures and largely ignored the WM. Thus the major advantage of our atlas is that it is unique in the combination of species and structure set that it aims to define.

Other advantages relate to the quality of the DTI-based template on which the atlas was drawn. Because the atlas was drawn on a template created from 271 individual subjects, regions could be defined smoothly and with greater detail than the resolution of the individual scans would allow. Moreover, the atlas reflects more of a true population average, making it more applicable to studies across populations than an atlas drawn from a single subject would be. Not only is it a template from a large population, but it is one that was made using cutting-edge tensor-based registration methods that have only become available in recent years. Using the full tensor information to create the atlas improves on the registration quality in a measurable way over FA or T2-W image registration [31]. It also allows any necessary DTI measures to be generated directly from the template tensors rather than registered from individuals. In this case the color-coded directional FA information was especially helpful in determining ROI boundaries, and the fact that the color map was derived directly from the template tensors increases confidence in its accuracy.

There are some limitations of the atlas. It was created using a large population template, but the original data was taken with only 12 directions and a relatively low resolution (0.625×0.625×2.5 mm^3^). The boundaries of the atlas may be refined and improved in future studies with data of higher spatial resolution, parallel imaging to reduce EPI distortions and more encoding directions, though the latter is not imperative for DTI.

Similar to the human WM atlas [8], some divisions are very subjective, especially in larger structures without clear tissue boundaries such as the corona radiata and corpus callosum. However, we avoided entirely arbitrary choices for boundaries and instead used all available information to make informed (albeit subjective) choices. The genu, body, and splenium of the corpus callosum, for example, were divided in a way that avoided separating a single structure into two “pieces” in the more lateral sagittal slices. The boundaries between the corona radiata and the nearby corpus callosum and internal capsule tracts were determined by using a combination of directional color (using color gradients to locate places where fibers changed direction as a good candidate for an ROI boundary) and FA intensity (visually locating local minima in FA intensity as possible places where one tract might end and another begin) information. Ultimately, these large tracts could be separated – or smaller tracts combined – at the end user’s discretion, and this would still require less effort than drawing a desired ROI by hand, while still maintaining the uniformity of reporting results based on atlas locations.

Differences in anatomy between the human and the macaque are responsible for some differences between this and the human atlas. For example, the fornix and anterior commissure are both relatively bigger in size in the rhesus in comparison to the human brain. In the rhesus, the temporal gyri are very vertical and the separation between superior, middle and inferior white matter is much clearer than in a human brain.

The current atlas also allows the study of differences in WM architecture between human and monkey. Comparative anatomy shows that both species have largely the same set of white matter bundles, however their proportions are different [85]–[90]. With the creation of this rhesus atlas, designed to be comparable to the ICBM-DTI-81 human atlas, one can potentially compare the ROI sizes in each atlas to analyze the proportions of key tracts between the two species. While the proposed template was designed to be consistent with the JHU template, other templates theoretically may provide more functionally- and/or tract- specific parcellations of the WM.

The atlas may be warped into the space of any study-specific template. In the example we have shown here, the alignment of the white matter tracts appears reasonable (Fig. 11). We generated manual ROIs of selected regions to compare quantitatively against the atlas-defined regions in our example. For each of the selected regions, Dice coefficients indicate strong agreement. Studies of automated image segmentation methods have shown Dice coefficients on the order of 0.8 in good segmentations, consistent with our results. (In cases of image irregularities such as tumors, the same segmentations have resulted in Dice coefficients as low as 0.49) [91]. The PTR had the poorest match, which is to be expected as it has peripheral components that twist and branch, making it a more difficult pathway to objectively identify on an FA map.

Since this WM atlas was created in the same space as the digitized Paxinos atlas, the two atlases may be used in conjunction in studies interested in both WM and GM, removing the need to calculate an additional warp or registration. This atlas will be made publicly available online and will serve as novel and valuable tool for the non-human primate neuroimaging community.
